# Allogeneic human neural stem cells for improved therapeutic delivery to peritoneal ovarian cancer

**DOI:** 10.1186/s13287-021-02226-8

**Published:** 2021-03-24

**Authors:** Rachael Mooney, Wafa Abidi, Jennifer Batalla-Covello, Hoi Wa Ngai, Caitlyn Hyde, Diana Machado, Asma Abdul-Majid, Yanan Kang, Mohamed Hammad, Linda Flores, Greg Copeland, Thanh Dellinger, Ernest Han, Jacob Berlin, Karen S. Aboody

**Affiliations:** 1 City of Hope Familian Sciences 1014A, Department of Developmental and Stem Cell Biology, Beckman Research Institute at City of Hope, 1500 East Duarte Road, Duarte, CA 91010 USA; 2 Irell & Manella Graduate School of Biological Sciences, Beckman Research Institute at City of Hope, 1500 East Duarte Road, Duarte, CA 91010 USA; 3 Department of Molecular Medicine, Beckman Research Institute at City of Hope, 1500 East Duarte Road, Duarte, CA 91010 USA; 4 Division of Gynecologic Surgery, Beckman Research Institute at City of Hope, 1500 East Duarte Road, Duarte, CA 91010 USA

**Keywords:** Neural stem cells, Ovarian cancer, Peritoneal metastases, Tumor tropism, Cell therapy, Drug delivery

## Abstract

**Background:**

Immortalized, clonal HB1.F3.CD21 human neural stem/progenitor cells (NSCs), loaded with therapeutic cargo prior to intraperitoneal (IP) injection, have been shown to improve the delivery and efficacy of therapeutic agents in pre-clinical models of stage III ovarian cancer. In previous studies, the distribution and efficacy of the NSC-delivered cargo has been examined; however, the fate of the NSCs has not yet been explored.

**Methods:**

To monitor NSC tropism, we used an unconventional method of quantifying endocytosed gold nanorods to overcome the weaknesses of existing cell-tracking technologies.

**Results:**

Here, we report efficient tumor tropism of HB1.F3.CD21 NSCs, showing that they primarily distribute to the tumor stroma surrounding individual tumor foci within 3 h after injection, reaching up to 95% of IP metastases without localizing to healthy tissue. Furthermore, we demonstrate that these NSCs are non-tumorigenic and non-immunogenic within the peritoneal setting.

**Conclusions:**

Their efficient tropism, combined with their promising clinical safety features and potential for cost-effective scale-up, positions this NSC line as a practical, off-the-shelf platform to improve the delivery of a myriad of peritoneal cancer therapeutics.

**Supplementary Information:**

The online version contains supplementary material available at 10.1186/s13287-021-02226-8.

## Background

Ovarian cancer is a deadly disease that afflicts approximately 22,000 women in the USA per year [1]. For patients with stage III ovarian cancer, in which tumors have metastasized to the abdominal cavity, the 5-year survival rate is only 34% [1]. Intraperitoneal (IP) chemotherapy confers a significant survival advantage in these patients, but its widespread use has been limited by its toxic side effects, which often prevent treatment completion. A targeted delivery system to concentrate therapeutics specifically at ovarian tumor sites could substantially enhance treatment efficacy and reduce undesirable side effects, improving quality of life [2]. Furthermore, standard of care includes invasive surgical debulking. This process only removes macroscopic tumors, leaving behind microscopic tumors. A targeted therapeutic delivery system with better distribution would help target those micro-metastases post-surgery.

Tumor-tropic cell carriers, which have a propensity to migrate to tumor sites, have shown promise as a tumor-targeted delivery system. For example, we and others have demonstrated that cell carriers afford advantages over free nanoparticle (NP) delivery in the IP cavity. Specifically, mesenchymal stem cells (MSCs), neural stem/progenitor cells (NSCs), T-cells, and macrophages can dramatically increase the efficiency and number of therapeutic NPs that localize to tumors in vitro and in vivo [3–7]. Cell-mediated tumor tropism is not passive, but rather an active, discriminating process mediated by a myriad of tumor-localized signals [8]*.* Because cell carriers are approximately 10 μm in diameter, they mainly deposit on the peritoneal surface rather than pass through it [9, 10]. Furthermore, cell carriers injected into the IP cavity can increase the retention of small (~ 100-nm) NPs from mere hours to several days [11].

Autologous MSCs, which have been the predominant cell type investigated for peritoneal therapeutic delivery [12, 13], can be isolated from a patient’s bone marrow or adipose tissues, expanded and modified ex vivo, and re-administered. However, there are major drawbacks to the use of MSCs, including that they are composed of heterogeneous cell populations, have poorly reproducible ex vivo loading capacities, and lose their tumor-homing properties after 5–6 passages [13]. Moreover, the amount and quality of MSCs that can be isolated depend critically on patient age and health status, and it can take 2 weeks after isolation to generate a sufficient number of cells for treatment [13]. In addition, it was recently reported that 20% of expanded MSCs had abnormal karyotypes [13], which is of deep concern as the confirmed non-tumorigenicity of any clinical stem cell therapy is of paramount importance. Consequently, although autologous MSCs may be feasible therapeutic delivery vehicles for smaller phase I trials, they represent an inefficient and poorly reproducible approach that will be difficult to pass through regulatory hurdles and meet the scale-up requirements necessary for phase II and III trials [13]. Therefore, developing a more clinically viable strategy to deliver therapeutic cargo selectively to tumor sites is critical.

In our previous work [14, 15], we investigated the use of an established tumor-tropic human clonal NSC line, HB1.F3.CD21, for targeted IP therapy in orthotopic mouse models of high-grade serous ovarian cancer, the most common histotype of stage III peritoneal disease. These NSCs are chromosomally and functionally stable over time and passage, HLA class II-negative, and their clinical safety, tumor tropism, and tumor-localized chemotherapy production in recurrent glioma patients have been demonstrated [16]. Importantly, this NSC line has established standard operating procedures for scaled-up production that are more efficient, reproducible, and economical than those used for autologous cells. Thus, the line can be expanded, modified, and banked as an “off-the-shelf” product, readily available for trials at multiple sites [17]. Although we previously demonstrated that the IP administration of these NSCs improves the delivery and therapeutic efficacy of drug-loaded NPs [14, 15] and oncolytic viruses [18] in ovarian metastases, additional studies are required to determine the efficiency of each administration, degree of tumor coverage, penetration of the tumor stroma, and potential unintended tumorigenic/immunogenic effects of these NSCs.

In the present study, we conduct comprehensive pharmacokinetic and biodistribution assessments to probe the efficiency, peritoneal distribution, tumorigenicity, and immunogenicity of this NSC-based therapeutic delivery platform. We investigate NSC tumor tropism in both immunodeficient and immunocompetent orthotopic mouse models of IP ovarian metastases. Using these models, we confirm our previous observation that NSCs localize only to tumor nodules and not to normal tissues [14, 18]. We also demonstrate that HB1.F3.CD21 NSCs target ovarian metastases with remarkable efficiency and selectivity, positioning this cell line as an excellent delivery platform to improve the therapeutic index of IP anticancer treatments.

## Methods

### Cell culture

The *v*-myc immortalized, clonal human HB1.F3.CD21 NSC line (approved by the Food and Drug Administration for human glioma clinical trials via local injection, Identifier: NCT01172964) was obtained from Dr. Seung Kim (University of British Columbia, Canada). The NSCs were further modified to produce NSC.eGFP.ffluc cells expressing green fluorescent protein (eGFP) and firefly luciferase (ffluc), as previously described [19]. The ID8 murine ovarian cancer line was obtained from Dr. Katherine Roby (University of Kansas). These cells were further modified using a PJ01668-eGFP-ffluc-epHIV7 lentiviral vector (159e6 TU/ML; VF0716) generously provided by Dr. Christine Brown (City of Hope) to produce ID8.eGFP.ffluc cells. Reporter gene expression and tumorigenicity were confirmed for the ID8.eGFP.ffluc line prior to study initiation (Supplementary Fig. 1). eGFP and ffluc-expressing OVCAR8 human ovarian cancer cells (OVCAR8.eGFP.ffluc) were generously provided by Dr. Carlotta Glackin (City of Hope). The NSC and ID8 cell lines were cultured in DMEM (Invitrogen) and the OVCAR8 cell line was cultured in RPMI basal media; all media were supplemented with 10% fetal bovine serum (Gemini Bio), 1% L-glutamine (Invitrogen), and 1% penicillin-streptomycin (Invitrogen). All cells were maintained at 37 °C in a humidified incubator (Thermo Electron Corporation) containing 6% CO_2_ and passaged using a 0.25% trypsin and EDTA solution (Invitrogen) when they reached 80% confluency, and media were changed every 2–3 days.

### In vivo NSC administration and tracking in ovarian cancer models

Mice were maintained under specific pathogen-free conditions in the City of Hope Animal Resource Center, an AAALAC-accredited facility. All procedures were reviewed and approved by the City of Hope Animal Care Committee. For our immunodeficient model, 6–8-week-old female nude mice (The Jackson Laboratory) were inoculated with 2 × 10^6^ OVCAR8.eGFP.ffluc cells via IP injection. For our immunocompetent model, 7–8-week-old female C57Bl/6 mice (B6, National Cancer Institute [NCI]) were inoculated with 5 × 10^6^ ID8.eGFP.ffluc cells via IP injection. After tumor development (3 weeks for OVCAR8, and 6 weeks for ID8), mice received NSCs (10,000–1 × 10^7^ total) labeled with lipophilic tracers (DiR; Thermo Fisher Scientific), 811-nm MUTAB-conjugated gold nanorods (AuNRs; Nanopartz) [14]). At select time points after administration of NSCs, mice underwent live-animal imaging (described below), then peritoneal lavage fluid was collected, as previously described [20], and tumors were harvested from major organs (liver, kidney, stomach, intestines, and mesentery) and processed for inductively coupled plasma mass spectrometry (ICP-MS) quantification or fluorescence imaging, as described below.

### Live-animal imaging

Tumor burden and NSC localization were evaluated via bioluminescence imaging. To determine if AuNRs affect NSC viability or tropism in vivo, OVCAR8.eGFP.ffluc tumor cells were used to inoculate nude mice and DiR-labeled NSCs with or without AuNRs were injected IP. To track NSC clearance in another experimental subset of mice, unlabeled tumor cells were used to inoculate nude mice, and DiR-labeled NSC.eGFP.ffluc cells were injected IP. Prior to imaging, mice were anesthetized by isoflurane (1.5 L/oxygen, 4% isoflurane) in an induction chamber and injected IP with D-luciferin substrate suspended in PBS at 4.29 mg/mouse. Mice were maintained under anesthesia in the chamber, and NSCs were imaged 7 min after luciferin injection using a SPECTRAL Ami X charge-coupled device camera coupled to Ami X image acquisition and analysis software. Light emission was measured over an integration time of 30 s.

### ICP-MS quantification of AuNRs

Concentrations of gold (AuNRs) in tumors and peritoneal lavage fluid were determined using an Agilent 8800 inductively coupled plasma triple quadrupole mass spectrometer. Briefly, each sample was digested with 70% HNO_3_ at 80 °C for 16 h. Samples were diluted with 2% HNO_3_ prior to injection into the mass spectrometer, and the detected signals were determined based on standard curves made using serial dilutions of gold (100 ppm) standard solutions (Spex Certiprep) in 1% HCl and 2% HNO_3_. To determine the percentage of AuNRs that localized to tumors vs. the lavage fluid, their concentrations were normalized to the concentrations detected in the original cell suspension that was injected into each mouse.

### Fluorescence imaging

Tumors were frozen in Tissue Tek OCT (Sakura Finetek USA) and sectioned on a Leica CM1510 S cryostat (Leica Biosystems). Sections (10 μm) were collected on positively charged slides (Thermo Fisher Scientific), immunostained for active caspase-3/7 (AB3626; Chemicon), counterstained with DAPI (1 μg/mL; Sigma), and imaged using a Zeiss Axio Observer Z1 fluorescence microscope (Zeiss Microscopy). For 3D histological reconstruction to visualize NSC penetration, a subset of tumors was sectioned into 10-μm-thick slices, imaged in their entirety at 200-μm intervals, and virtually reconstructed using Reconstruct software [21].

### Mixed lymphocyte reaction

To assess the immunogenicity of the NSCs in vitro, peripheral blood mononuclear cells (PBMCs) were incubated with NSCs and their degranulation measured, as previously described [22]. Briefly, PBMCs were isolated from seven healthy volunteers, using standard Ficoll density gradient centrifugation, and cryopreserved. Thawed aliquots of PBMCs were co-cultured with NSCs (1:1 final ratio), and FITC-conjugated antibodies against CD107a and CD107b (BD Pharmingen) were added to the cultures, followed by a monensin-containing protein transport inhibitor (GolgiStop; BD Biosciences). Cells were then stained with antibodies against CD3, CD4, CD56, CD16, CD14, and CD19 (BD Pharmingen) and analyzed using a Gallios flow cytometer (Beckman Coulter). Monocytes and B cells that expressed CD14 and CD19 were excluded. PBMCs stimulated with phorbol myristate acetate (PMA; 50 ng/ml) and phytohemagglutinin (PHA; 1 μg/ml) served as positive controls.

### Flow cytometric analysis of PD-L1 and CD47 expression

Trypsinized NSCs were stained with PE/Dazzle594-conjugated antibodies against human PD-L1 (clone 29E2A3) and CD47 (clone CC2C6) and their respective isotype controls (BioLegend). All flow samples were acquired using a Guava EasyCyte flow cytometer (Millipore). Histograms were generated using FlowJo (Tree Star).

### Tumorigenicity

To determine if IP injections of NSCs altered tumor progression in vivo, nude mice were inoculated IP with OVCAR8.eGFP.ffluc cells. After allowing tumors to progress unaltered for 1 or 3 weeks (early and late tumor stages, respectively), mice received IP injections of 2 × 10^7^ NSCs twice a week for 3 weeks. After the last week of treatment, mice were euthanized and tumors harvested, pooled, and weighed in pre-tared tubes.

### Statistical analysis

Data are presented as mean ± SEM, and statistical significance (*p* < 0.05) was determined using two-tailed Student’s *t*-tests.

## Results

### AuNR-based tracking of NSC tumor tropism within the peritoneal cavity

**Fig. 1:**
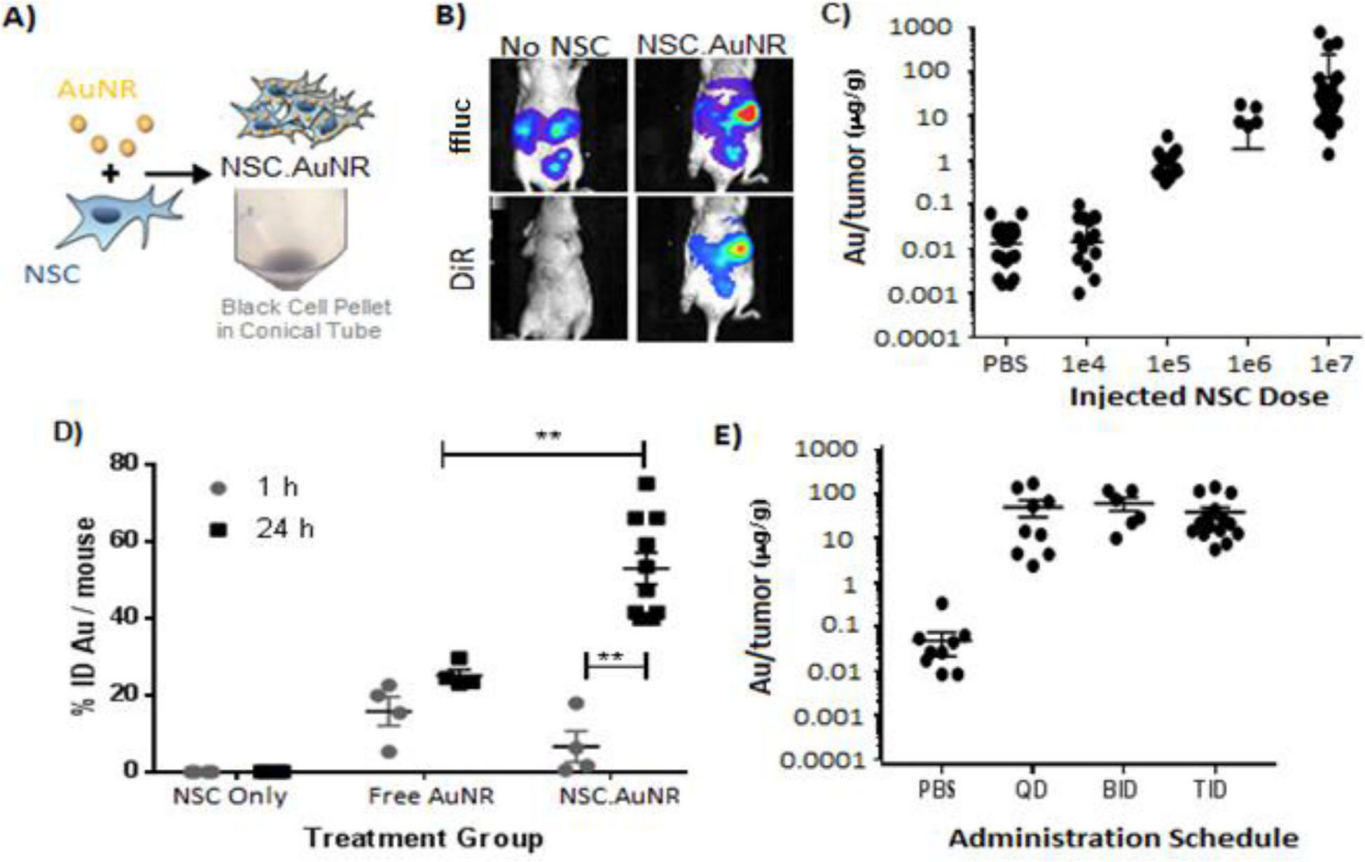
AuNR-based monitoring of HB1.F3.CD21 NSC tropism to peritoneal metastases. **a** Schematic showing AuNR-loaded NSCs (NSC.AuNRs), which are visibly black at the bottom of the conical tube shown in the photograph. **b** Fluorescent and bioluminescent images confirming that DiR-labeled NSC.AuNRs (bottom panel) co-localize with ffluc-expressing tumors (top panel). Nude mice were inoculated with 2 × 10^6^ OVCAR8.eGFP.ffluc tumor cells, and NSC.AuNRs, dual-labeled with DiR to track their distribution, were injected 3 weeks later. Images were acquired 1 h after IP NSC.AuNR injection. **c**–**e** ICP-MS quantification of AuNR levels within IP metastases. **c** ICP-MS quantification of tumor-localized NSC.AuNRs in a titration experiment demonstrating the limits and linear dose-response relationship of NSC.AuNR detection within the IP cavity. 1e7 dose represents 5 mice (30 tumors); 1e6 doses represents 1 mouse (5 tumors); 1e5 dose represents 3 mice (16 tumors); 1e4 and PBS dose represents 3 mice (15 tumors). **d** AuNR quantification as a percentage of signal in the injected dose [%ID] of either free AuNRs or NSC.AuNRs at 1 and 24 h after injection. **p < 0.01. For the 1-h data series, all groups represent *n* = 4 mice; for the 24-h data series, the NSC only and free AuNR groups represent *n* = 4 mice, and the NSC-AuNR group represents *n* = 10 mice. **e** ICP-MS quantification of 1 × 10^7^ NSC.AuNRs total, administered over 24 h via one (QID), two (BID), or three (TID) injections. The PBS group represents 3 mice (12 tumors); QID represents 3 mice (14 tumors); BID represents 2 mice (12 tumors); TID represents 3 mice (15 tumors)

In mouse models, tumor cells inoculated into the peritoneal cavity are carried by the physiological movement of the peritoneal fluid to seed the greater omentum, followed by the serosal surfaces of the liver, kidney, intestines, diaphragm, and peritoneum. The tumors that are formed consist of small (0.5–5-mm) nodules scattered over the peritoneal surface. Thus, it was necessary to identify a tracking method that is sensitive enough to quantify the presence of NSCs at individual nodules. In preliminary studies, we found that the signal afforded by membrane dyes and fluorescent gene markers was suboptimal for the sensitive quantification of NSCs localized to IP tumors (Supplemental Fig. 3). Instead, we labeled NSCs with internalized AuNRs (Fig. 1a), which provide an NSC-specific signal that can be assessed quantitatively with high sensitivity using ICP-MS.

We previously showed that AuNRs do not impair NSC viability or tropism in vitro [23, 24]. Here, we utilized live-animal imaging to demonstrate that DiR-stained NSCs labeled with AuNRs are able to migrate towards tumors within the peritoneal setting in vivo (Fig. 1b). We then validated the detection method by injecting different doses of AuNR-loaded NSC varied from 1 × 10^4^ to 1 × 10^7^. The minimum number of NSC.AuNRs required to produce measurable AuNR levels in harvested tumors (pooled) was about 100,000 (Fig. 1c), and detection was dose-responsive up to an injected dose of 1 × 10^7^ NSCs, which is the maximum practical dose based on scale-up considerations for human trials (Fig. 1c).

To determine the efficiency of NSC.AuNR migration to IP tumors, we quantified the AuNR content within suspensions of 1 × 10^7^ NSCs (total dose), which were then injected into the peritoneal cavities of nude mice bearing OVCAR8 tumors. Tumors were harvested at 1 and 24 h after NSC.AuNR injection to assess their Au content. ICP-MS analysis revealed that 60% ± 20% of the injected dose was localized to the tumors by 24 h (Fig. 1d). To confirm that the tumor-localized AuNR signal could be attributed to NSCs, we also evaluated control mice injected with a matching dose of free AuNRs and observed a signal 52 ± 13% lower than that of the NSC.AuNRs at 24 h.

We next tested if NSC localization to tumor sites could be enhanced by splitting the dose of 1 × 10^7^ NSC.AuNRs into two or three injections administered over a 24-h period (Fig. 1e). We found no significant differences in the quantity of NSC.AuNRs localized to the tumor when they were administered as a single bolus dose rather than in multiple injections.

### Kinetics of NSC tropism and cargo delivery

**Fig. 2:**
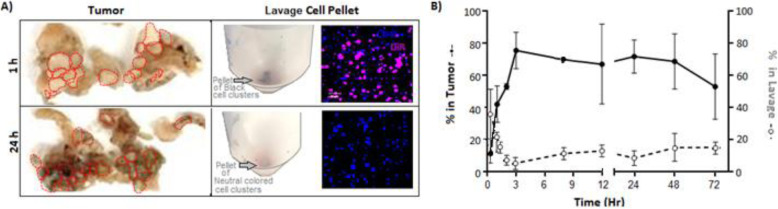
NSC tropism kinetics. **a** Photographs of ovarian tumor nodules (left panel, outlined in red) and centrifuged lavage cell pellets (center panel) after IP administration of 1 × 10^7^ DiR-labeled NSC.AuNRs, demonstrating the localization of black NSCs in the lavage fluid at 1 h and in tumors at 24 h. The lavage cell pellets were also imaged with fluorescence microscopy (right panel) to visualize the number of DiR-labeled NSC.AuNRs (magenta) at each time point. Anti-CD45 antibodies were used to visualize macrophages (blue). **b** ICP-MS quantification of NSC-delivered AuNRs within ovarian metastases (solid lines) and peritoneal lavage fluid (dashed lines) over a 72-h period following IP NSC administration into tumor-bearing nude mice. Each point represents the mean ± standard deviation for *n* = 3 mice/time point

The migration of NSCs from the peritoneal cavity to the tumors could be observed macroscopically, because the AuNRs are visibly black. This resulted in localized NSCs altering the tumor appearance from white to grayish within 24 h (Fig. 2a). Conversely, the pellet of cells harvested from the lavage fluid was black soon after IP administration of NSC.AuNRs but white within 24 h (Fig. 2a). To further evaluate NSC tropism kinetics, we quantified Au levels within tumors and peritoneal lavage fluid collected at several time points after IP administration of NSC.AuNRs in OVCAR8 tumor-bearing nude mice. ICP-MS analysis demonstrated that 60 ± 20% of the NSC dose localized to tumors within 2–3 h after IP administration (Fig. 2b). At all tested time points (1–72 h), 70–80% of the injected NSCs were accounted for in either the lavage fluid or the tumors.

### NSC tumor coverage and biodistribution

**Fig. 3:**
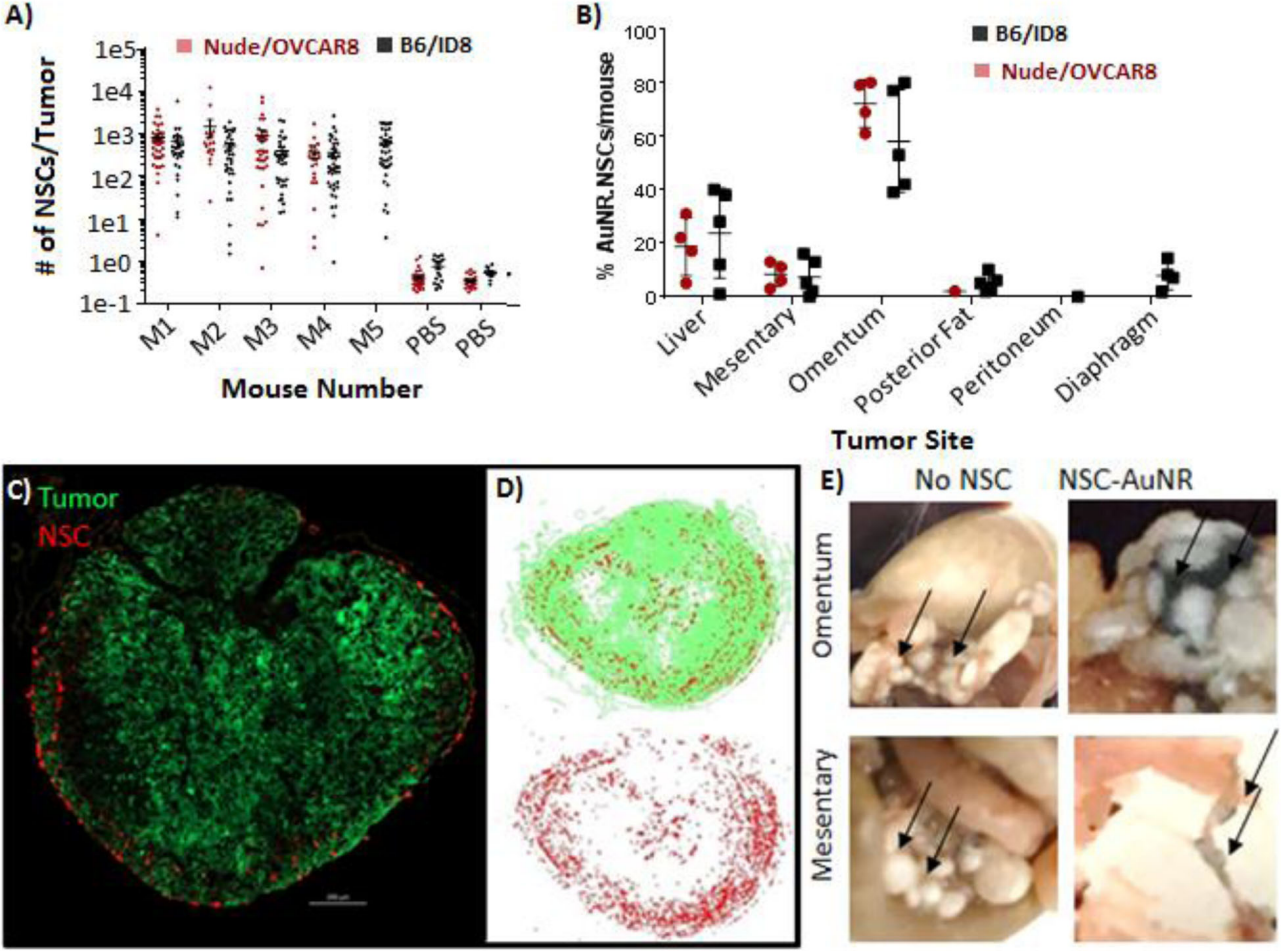
NSC biodistribution within the IP cavity. **a**, **b** ICP-MS quantification of NSC tumor tropism efficiency within immunodeficient (nude/OVCAR8) and immunocompetent (B6/ID8) mice. **a** The absolute number of NSCs localized to individual macroscopic tumors was determined based on a measurement of 5 pg AuNR/NSC. Data from nude mice represent *n* = 4 mice, 113 tumors for the NSC group, and *n* = 2 mice, 49 tumors for the PBS group. Data from the B6 mice represent *n* = 5 mice, 169 tumors for the NSC group, and *n* = 2 mice, 28 tumors for the PBS group. **b** Localization of NSC.AuNRs in tumors associated with specific organs, expressed as a percentage of the total number of tumor-associated NSCs. Each data point is the summed percentage present in each mouse analyzed in **a**. For the nude mice, data represents *n* = 4 mice (20 liver tumors, 29 mesentary tumors, 51 omental tumors, 12 lower fat tumors, no peritoneal tumors). For the B6 mice, data represents *n* = 5 mice (18 liver tumors, 39 mesentary tumors, 65 omental tumors, 25 lower fat tumors, 14 peritoneal tumors). **c**–**e** Distribution of IP-administered NSCs in the tumor parenchyma and peritumoral stroma. **c** Representative fluorescence microscopy image of an ovarian tumor nodule (green) and surrounding NSCs (red). **d** Flattened (top-down) 3D rendering of the distribution of NSCs throughout a single tumor nodule in **c**. **e** Photographs representative of tumors found on all organs demonstrating the presence of visibly black tumor-associated NSC.AuNRs localized to peritumoral stroma, 24 h after injection into OVCAR8 tumor-bearing nude mice

To assess the extent of NSC tumor coverage, we isolated individual IP tumor nodules and used ICP-MS to determine the number of NSCs contained therein. After IP administration of 1 × 10^7^ NSC.AuNRs to immunodeficient (nude) or immunocompetent (B6) mice bearing peritoneal ovarian metastases (OVCAR8 and ID8, respectively), more than 95% of tumor metastases were found to contain at least 10 NSCs, with each tumor containing a median of 425 NSCs (Fig. 3a). We also evaluated the distribution of NSC.AuNRs across tumors associated with specific organs. AuNR levels were particularly high in the omentum (Fig. 3b), the primary site of ovarian cancer metastases in patients [25], which accounted for approximately 40% of the collected tumor tissue by mass. To evaluate the distribution of NSCs within individual tumor nodules, we performed serial cryosectioning, fluorescence imaging, and 3D tumor reconstruction. The NSCs were generally distributed at the peritumoral stroma, with some penetration into the tumor parenchyma if there was also stromal infiltration (Fig. 3c, d). Consistent with our previous findings [14], no obvious off-target distribution was observed in any cryosectioned tissue samples (Supplementary Fig. 2). NSC.AuNR distribution to the tumor-associated stroma was observable macroscopically as a grayish-colored tissue connecting individual tumor metastasis (Fig. 3e).

### NSC immunogenicity and tumorigenicity

**Fig. 4:**
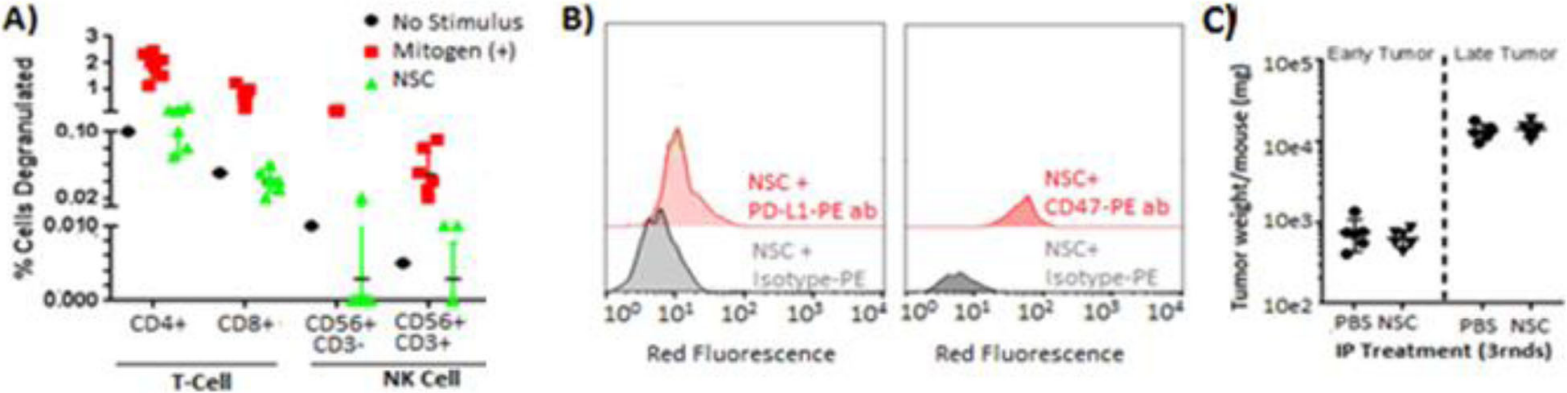
Immunogenicity of NSCs. **a** Fractions of CD107a/b-positive cytotoxic T-lymphocytes (CD3+, CD8+), T-helper lymphocytes (CD3+, CD4+), and natural killer cells (CD56+, CD3− and CD56+, CD3+) in response to NSC exposure. The City of Hope Clinical Immunobiology Correlative Studies Laboratory obtained whole blood from normal human donors (*n* = 7), isolated PBMCs via Ficoll density gradient centrifugation, then co-cultured them with NSCs in the presence of antibodies against CD107a/b. Positive control PBMCs were exposed to PMA and PHA. After a 5-h incubation period, flow cytometry was performed, with compensation for non-viable cells and isotype controls. **b** Flow cytometric quantification of PD-L1- and CD47-positive NSCs. **c** Pooled weights of macroscopic OVCAR8 tumors, as well as their associated stroma, in *n* = 6 nude mice after three weekly rounds of 2 × 10^7^ NSCs given 2×/week (for a combined total of 12 × 10^7^ NSCs). Treatment started after 1 week or 3 weeks after tumor cell inoculation to evaluate early- and late-stage tumors, respectively

HB1.F3.CD21 NSCs are generally considered to have negligible immunogenicity. They inherently express low levels of MHC class I antigens and undetectable levels of MHC class II antigens. We observed negligible immunological recognition of parental NSCs by PBMCs in vitro (Fig. 4a). The NSCs also had low expression of PD-L1 and CD47 (Fig. 4b), presumably reducing their visibility to the innate and adaptive immune systems, respectively. In addition, we demonstrated that bi-weekly IP administrations of 2 × 10^7^ NSCs (greater than the maximum clinical dose) did not promote ovarian tumor growth in immunodeficient (nude) mice bearing early- or late-stage OVCAR8 tumors (Fig. 4c).

## Discussion

Collectively, our results demonstrate the remarkable extent to which HB1.F3.CD21 NSCs rapidly and selectively home to an overwhelming majority of peritoneal metastases. Here, we discuss our major findings in the context of current literature.

### IP-administered NSCs localize efficiently to peritoneal metastases

Although IP-administered NSCs have the potential to migrate anywhere on the vast peritoneal surface area (1.7 m^2^), our ICP-MS-based quantification of NSC-associated AuNRs unexpectedly showed that an impressive 70–80% of NSCs localized to tumors, reaching up to 95% within 3 h after injection (Figs. 2b and 3a). One possible explanation for this tumor-specific localization is that the healthy mesothelium is protected by a layer of anti-adhesive proteoglycans, whereas tumor disruptions expose the underlying basement membrane, revealing extracellular matrix substrates (e.g., collagen I/IV, fibronectin, hyaluronan, and laminin) that can be bound by CD44 [26] and α_5_β_1_ integrins [27] expressed by the HB1.F3.CD.21 NSCs. Because this study focused only on intra-abdominal tumors and peritoneal lavage fluid, we were unable to account for the distribution of approximately 20% of injected NSCs. A minority of these NSCs may have been cleared from the abdomen through either the general circulation or lymphatic systems.

The behavior of NSCs, including their pharmacokinetic and biological properties, can be affected by the mode of administration [28–30]. While intravenous administration offers several clinical advantages including ease of delivery and access to systemic metastasis, local IP administration is often pursued in the ovarian cancer setting to increase the bioavailability of treatments at the target site. For example, a landmark clinical trial reported a significant survival benefit for ovarian cancer patients who had undergone a combination of intravenous (IV) and intraperitoneal (IP) cisplatin and paclitaxel as compared to IV paclitaxel and cisplatin alone [31]. Local IP administration was chosen for the current study because most stem cell-based therapies that have progressed to late-stage clinical trials have used local administration (i.e., intracranial, intrathecal, intralesional, and endocardial) [31]. Furthermore, we have previously demonstrated that when NSCs are administered IV rather than IP, little to no NSCs are observed in tumors in the IP cavity [14]. Systemic administration of stem cells still has key challenges including the instant blood-mediated inflammatory reaction (triggers coagulation), the pulmonary bypass barrier, and insufficient residence time at the target site [32].

We used the unconventional method of quantifying endocytosed AuNRs to monitor NSC tropism in order to overcome the weaknesses of existing cell-tracking technologies. Although the NSCs are engineered to express both eGFP and ffluc, given the resolution limits of live-animal imaging, these markers do not permit sensitive, quantitative NSC detection. Live-animal imaging for ovarian tumor models can also be particularly troublesome as the depth and location of the tumors around the IP organs can limit the signal detection. Furthermore, tumor and stromal DNA/cell counts overwhelm NSC-specific signals, so the number of NSCs present in tumor tissues is not reliably discernable using PCR or FACS. In addition, membrane dyes cannot be used without a complementary approach due to the possibility of dye transfer from injected cells to host cells as well as the possibility of photo-bleaching and fluorophore instability after fixative methods [33]. In contrast, inorganic NP trackers provide a better balance of sensitivity, dynamic range, and stability for assessing the distribution of IP-administered NSCs as there is no background signal to hinder quantification. Importantly, this method also enables the macroscopic observation of NSC migration, as well as the ability to assess NSC cargo delivery (Figs. 1a and 2a).

### NSCs provide an efficient therapeutic cargo delivery system

Our results demonstrate that the impressive tumor tropism of NSCs to IP metastases may significantly advance peritoneal chemotherapy by guiding the delivery of pre-loaded therapeutic cargo. We observed that, whereas the localization of NPs to tumors is low when they are delivered freely (perhaps because they are engulfed by peritoneal or tumor-associated macrophages), their tumor localization was significantly (more than 60%) greater when they were delivered within NSCs (Fig. 1d). We believe that this improvement can be generalized to other free vs. NSC-delivered therapeutic payloads. For example, we have previously reported improved NSC-mediated drug delivery of two standard-of-care chemotherapeutic drugs, cisplatin [14] and paclitaxel (PTX) [15] to peritoneal metastases, as well as two oncolytic viruses [18, 34]. This delivery system can not only improve tumor localization, but also potentially reduce toxicity and prolong the release of therapeutic reagent.

One critical insight yielded by the current study was that the NSCs localized primarily to the peritumoral stroma, with limited penetration into the tumor parenchyma (Fig. 3e). Thus, it is possible that improving the delivery of a drug to tumor nodules using NSCs may not be sufficient to improve its efficacy if other parameters dominate the response to treatment. For example, NSC-mediated drug delivery only improved the therapeutic efficacy of PTX [15] but not cisplatin (unpublished data). Other important parameters include diffusion limits [cisplatin (200 cell layers) vs PTX (80 layers) [35]]; peritoneal-to-plasma area under the concentration-time curve ratio [cisplatin (7.8–21) [36, 37] vs PTX (853) [38]]; treatment schedule [cisplatin (slow release, treated weekly) vs PTX (burst release, treated bi-weekly); and impaired biological activity of cisplatin after release from NSCs, microenvironmental priming by PTX that improves drug penetration or immune stimulation upon repeated dose cycles [39–43]. It will be important to consider and address these factors when selecting and developing therapeutic cargo for effective NSC delivery. Conversely, co-localization of NSCs to the tumor stroma provides an advantage in targeting the tumor stroma components that support tumor growth and metastasis [44]. This can also be exploited to deliver immunotherapeutic reagents in a targeted setting, preventing systemic disadvantages of immunotherapy.

### NSC safety considerations

Our result shows that HB1.F3.CD21 NSCs are non-tumorigenic in the peritoneal setting (Fig. 4c), consistent with our current clinical data in the glioma setting, as patients in phase I trials of allogeneic NSC-mediated enzyme/prodrug and CRAd-S-pk7 treatments have tolerated multiple intracranial administrations without adverse events or evidence of secondary tumorigenicity [16]. In stark contrast, MSCs have been reported to functionally engraft into peritoneal organs [45, 46] and can promote ovarian tumor growth by inducing the expression of IL-1, associating with macrophages, and transforming into carcinoma-associated MSCs [47]. IP MSC administration has also been shown to increase pro-inflammatory cytokines in mice, triggering such dramatic omental immune cell influx that the organ doubled in weight [48]. The results presented here show that NSCs induce negligible immunological recognition in vitro (Fig. 4a); however, further investigation of the potential immunomodulatory effects of NSCs within the IP cavity will be important within the context of future therapeutic efficacy studies, particularly for repeated NSC administrations.

Although autologous mesenchymal stem cells have also been used clinically to improve viral delivery to ovarian metastases [12], our allogeneic, off-the-shelf cell NSC line has essential practical advantages that enable cost-effective scale-up and greater reproducibility between patients. Following extensive characterization of the HB1.F3.CD NSC line [49], we have already pioneered the clinical translation of genetically modified NSCs for four cancer (glioma and neuroblastoma) therapies [50]. We have experience expanding, modifying, and banking these NSCs as “off-the shelf” products, readily available for large trials at multiple sites. As NSC-based therapies progress into the clinic for targeted cancer treatment within the peritoneal setting, understanding the pharmacokinetics of the NSCs administered into this setting is important prior to performing IND-enabling studies involving NSCs modified with a therapeutic. Our plan is to streamline the translation of our existing therapeutic approaches to first-in-human phase I trials for stage III ovarian cancer patients who fail surgical and chemotherapy standard of care, taking advantage of the Good Manufacturing Practice (GMP) standard operating procedures (SOPs) established for our ongoing clinical studies in other cancer settings.

## Conclusion

This allogeneic, immortalized, GMP-grade NSC line has significant practical and economic advantages over autologous cell carriers. It provides a non-tumorigenic, “off-the-shelf” platform that is readily available for modification, scale-up, and banking. Furthermore, it is amenable to the delivery of a broad array of therapeutic payloads within the peritoneal cavity. Our translational interest is in developing these cells for improved delivery of drug-loaded NPs, oncolytic viruses, and other promising therapeutic cargo, including bispecific T-cell engagers, small interfering RNA, and antibodies to patients with abdominal metastatic disease. Our long-term vision is to introduce cargo-loaded NSCs after surgical debulking. Surgical trauma creates disruptions to the patient’s mesothelial layer which serve as privileged sites for cancer cell attachment [51, 52]. However, we anticipate that NSCs will also be attracted to these regions and may be able to deliver a therapeutic dose strong enough to eliminate the attached tumor cells, thus preventing new tumor development.

## Supplementary Information

**Additional file 1: Supplementary Figure 1**. Murine ID8 ovarian cancer cell line modified to stably express green fluorescent protein (eGFP) and firefly luciferase (ffluc). (A): Phase and fluorescence microscopy images of eGFP-positive ID8 (ID8.eGFP.ffluc) cells five days post-infection. Scale bar = 50 μm. (B): Representative histogram of ID8.eGFP.ffluc cells quantified by flow cytometry 15 days after infection. (C): Bioluminescent images confirming tumor engraftment after peritoneal administration of 5 × 10^6^ ID8.eGFP.ffluc cells into immunocompetent C57Bl/6 mice, color scale bar shown in relative light units. (D): Representative photograph demonstrating the development of ascites in a C57Bl/6 mouse, two months after inoculation with ID8.eGFP.ffluc cells. (E): Ex vivo photograph of the peritoneal wall harvested from a C57Bl/6 mouse inoculated with ID8.eGFP.ffluc cells.

**Additional file 2: Supplementary Figure 2**. Neural stem cells tropism to peritoneal ovarian cancer metastasis. Representative fluorescence images of neural stem cells labeled with either magenta DiD (A-C) or fluorescent orange (D-F) or red nanoparticles (G-I). Neural stem cells demonstrate good distribution in tumor but not in adjacent normal tissues (liver, intestine, kidney, omentum, stomach, pancreas or spleen). (2 million NSCs in 200uL PBS injected i.p. on Day 38; then harvested 4 days post-NSC injection). Scale bars = 1000 μm and applies to all images.

**Additional file 3: Supplementary Figure 3**. NSC clearance kinetics. (A) Retention and viability of DiR-labeled NSC.eGFP.ffluc cells over a two-week period following IP injection into *n* = 4 tumor-bearing nude mice (solid line, DiR fluorescence; dotted line, ffluc expression). Once localized to tumors, the NSCs remained present for at least 3 days, according to steady NSC-associated DiR signals. However, NSC-specific firefly luciferase expression decrease quickly post-transplantation. (B) Fluorescent images of sectioned tumors obtained either 1 day (D1) or 11 days (D11) after IP injection of NSCs (magenta). Anti-caspase-3/7 antibodies were used to visualize apoptosis. DiR-labeled NSCs were ~ 30% positive for capsase-3/7 on both day 1 (35.0%) and day 11 (32.1%). Data represents % caspase + DIR-labeled NSCs observed in 3 stained slides of tumor sections over which 8 (Day 1) or 18 (Day 11) representative fields of view were quantified using ImageJ software.

## Data Availability

All data generated or analyzed during this study are included in this published article [and its supplementary information files].

## Abbreviations

AuNR: Gold nanorod
NSC: Neural stem cell
MSC: Mesenchymal stem cell
RNA: Ribonucleic acid
DNA: Deoxyribonucleic acid
NP: Nanoparticle
GMP: Good Manufacturing Practice
IL-1: Interleukin 1
PTX: Paclitaxel
PCR: Polymerase chain reaction
FACS: Fluorescence-activated cell sorting
IP: Intraperitoneal
eGFP: Green fluorescent protein
ffluc: Firefly luciferase
ICP-MS: Inductively coupled plasma mass spectrometry
MHC: Major histocompatibility complex
PBMC: Peripheral blood mononuclear cells
HLA: Human leukocyte antigen

## References

[1] Lengyel E (2010). Ovarian cancer development and metastasis. Am J Pathol.

[2] Ovarian cancer. Nat Rev Dis Primers. 2016 Aug 25;2:16062. 10.1038/nrdp.2016.62 27558277

[3] Huang B, Abraham WD, Zheng Y, Bustamante López SC, Luo SS, Irvine DJ (2015). Sci Transl Med.

[4] Stephan MT, Moon JJ, Um SH, Bershteyn A, Irvine DJ (2010). Therapeutic cell engineering with surface-conjugated synthetic nanoparticles. Nat Med.

[5] Choi M-R, Stanton-Maxey KJ, Stanley JK, Levin CS, Bardhan R, Akin D (2007). A cellular Trojan Horse for delivery of therapeutic nanoparticles into tumors. Nano Lett.

[6] Li L, Guan Y, Liu H, Hao N, Liu T, Meng X (2011). Silica nanorattle-doxorubicin-anchored mesenchymal stem cells for tumor-tropic therapy. ACS Nano.

[7] Mooney R, Weng Y, Garcia E, Bhojane S, Smith-Powell L, Kim SU (2014). Conjugation of pH-responsive nanoparticles to neural stem cells improves intratumoral therapy. J Control Release.

[8] Kendall SE, Najbauer J, Johnston HF, Metz MZ, Li S, Bowers M (2008). Neural stem cell targeting of glioma is dependent on phosphoinositide 3-kinase signaling. Stem Cells.

[9] Mirahmadi N, Babaei MH, Vali AM, Dadashzadeh S (2010). Effect of liposome size on peritoneal retention and organ distribution after intraperitoneal injection in mice. Int J Pharm.

[10] Lu Z, Tsai M, Lu D, Wang J, Wientjes MG, Au JL-S (2008). Tumor-penetrating microparticles for intraperitoneal therapy of ovarian cancer. J Pharmacol Exp Ther.

[11] De Smet L, Ceelen W, Remon JP, Vervaet C (2013). Optimization of drug delivery systems for intraperitoneal therapy to extend the residence time of the chemotherapeutic agent. ScientificWorldJ..

[12] Mader EK, Maeyama Y, Lin Y, Butler GW, Russell HM, Galanis E (2009). Mesenchymal stem cell carriers protect oncolytic measles viruses from antibody neutralization in an orthotopic ovarian cancer therapy model. Clin Cancer Res.

[13] Mader EK, Butler G, Dowdy SC, Mariani A, Knutson KL, Federspiel MJ (2013). Optimizing patient derived mesenchymal stem cells as virus carriers for a phase I clinical trial in ovarian cancer. J Transl Med.

[14] Cao P, Mooney R, Tirughana R, Abidi W, Aramburo S, Flores L (2017). Intraperitoneal administration of neural stem cell-nanoparticle conjugates targets chemotherapy to ovarian tumors. Bioconjug Chem.

[15] Tiet P, Li J, Abidi W, Mooney R, Flores L, Aramburo S (2019). Silica coated paclitaxel nanocrystals enable neural stem cell loading for treatment of ovarian cancer. Bioconjug Chem.

[16] Portnow J, Synold TW, Badie B, Tirughana R, Lacey SF, D’Apuzzo M (2017). Neural stem cell-based anticancer gene therapy: a first-in-human study in recurrent high-grade glioma patients. Clin Cancer Res.

[17] Tirughana R, Metz MZ, Li Z, Hall C, Hsu D, Beltzer J (2018). GMP production and scale-up of adherent neural stem cells with a quantum cell expansion system. Mol Ther Methods Clin Dev.

[18] Mooney R, Majid AA, Batalla-Covello J, Machado D, Liu X, Gonzaga J (2019). Enhanced delivery of oncolytic adenovirus by neural stem cells for treatment of metastatic ovarian cancer. Mol Ther Oncolytics..

[19] Cheng Y, Morshed R, Cheng S-H, Tobias A, Auffinger B, Wainwright DA (2013). Nanoparticle-programmed self-destructive neural stem cells for glioblastoma targeting and therapy. Small..

[20] Ray A, Dittel BN. Isolation of mouse peritoneal cavity cells. J Vis Exp. 2010;(35):1488. 10.3791/1488. 10.3791/1488 PMC3152216 20110936

[21] Fiala JC (2005). Reconstruct: a free editor for serial section microscopy. J Microsc.

[22] Metz MZ, Gutova M, Lacey SF, Abramyants Y, Vo T, Gilchrist M (2013). Neural stem cell-mediated delivery of irinotecan-activating carboxylesterases to glioma: implications for clinical use. Stem Cells Transl Med.

[23] Mooney R, Roma L, Zhao D, Van Haute D, Garcia E, Kim SU (2014). Neural stem cell-mediated intratumoral delivery of gold nanorods improves photothermal therapy. ACS Nano.

[24] Schnarr K, Mooney R, Weng Y, Zhao D, Garcia E, Armstrong B (2013). Gold nanoparticle-loaded neural stem cells for photothermal ablation of cancer. Adv Healthc Mater.

[25] Sodek KL, Murphy KJ, Brown TJ, Ringuette MJ (2012). Cell-cell and cell-matrix dynamics in intraperitoneal cancer metastasis. Cancer Metastasis Rev.

[26] Ahmed AU, Thaci B, Tobias AL, Auffinger B, Zhang L, Cheng Y (2013). A preclinical evaluation of neural stem cell-based cell carrier for targeted antiglioma oncolytic virotherapy. J Natl Cancer Inst.

[27] Ziu M, Schmidt NO, Cargioli TG, Aboody KS, Black PM, Carroll RS (2006). Glioma-produced extracellular matrix influences brain tumor tropism of human neural stem cells. J Neuro-Oncol.

[28] Antunes MA, Abreu SC, Cruz FF, Teixeira AC, Lopes-Pacheco M, Bandeira E (2014). Effects of different mesenchymal stromal cell sources and delivery routes in experimental emphysema. Respir Res.

[29] Eggenhofer E, Benseler V, Kroemer A, Popp FC, Geissler EK, Schlitt HJ (2012). Mesenchymal stem cells are short-lived and do not migrate beyond the lungs after intravenous infusion. Front Immunol.

[30] O’Cearbhaill ED, Ng KS, Karp JM (2014). Emerging medical devices for minimally invasive cell therapy. Mayo Clin Proc.

[31] Armstrong DK, Bundy B, Wenzel L, Huang HQ, Baergen R, Lele S (2006). Intraperitoneal cisplatin and paclitaxel in ovarian cancer. N Engl J Med.

[32] Markiewski MM, Nilsson B, Ekdahl KN, Mollnes TE, Lambris JD (2007). Complement and coagulation: strangers or partners in crime?. Trends Immunol.

[33] Lassailly F, Griessinger E, Bonnet D (2010). “Microenvironmental contaminations” induced by fluorescent lipophilic dyes used for noninvasive in vitro and in vivo cell tracking. Blood..

[34] Hammad M, Cornejo YR, Batalla-Covello J, Majid AA, Burke C, Liu Z (2020). Neural stem cells improve the delivery of oncolytic chimeric orthopoxvirus in a metastatic ovarian cancer model. Mol Ther Oncolytics.

[35] Los G, Mutsaers PH, Lenglet WJ, Baldew GS, McVie JG (1990). Platinum distribution in intraperitoneal tumors after intraperitoneal cisplatin treatment. Cancer Chemother Pharmacol.

[36] Carlier C, Mathys A, De Jaeghere E, Steuperaert M, De Wever O, Ceelen W (2017). Tumour tissue transport after intraperitoneal anticancer drug delivery. Int J Hyperth.

[37] Heldin C-H, Rubin K, Pietras K, Ostman A (2004). High interstitial fluid pressure - an obstacle in cancer therapy. Nat Rev Cancer.

[38] Levine EA, Ceelen WP (2015). Intraperitoneal cancer therapy : principles and practice.

[39] Kampan NC, Madondo MT, McNally OM, Quinn M, Plebanski M (2015). Paclitaxel and its evolving role in the management of ovarian cancer. Biomed Res Int.

[40] Kuh HJ, Jang SH, Wientjes MG, Weaver JR, Au JL (1999). Determinants of paclitaxel penetration and accumulation in human solid tumor. J Pharmacol Exp Ther.

[41] Jang SH, Wientjes MG, Au JL (2001). Enhancement of paclitaxel delivery to solid tumors by apoptosis-inducing pretreatment: effect of treatment schedule. J Pharmacol Exp Ther.

[42] Lu D, Wientjes MG, Lu Z, Au JL-S (2007). Tumor priming enhances delivery and efficacy of nanomedicines. J Pharmacol Exp Ther.

[43] Jang SH, Wientjes MG, Au JL (2001). Determinants of paclitaxel uptake, accumulation and retention in solid tumors. Investig New Drugs.

[44] Yin M, Li X, Tan S, Zhou HJ, Ji W, Bellone S (2016). Tumor-associated macrophages drive spheroid formation during early transcoelomic metastasis of ovarian cancer. J Clin Invest.

[45] Wilson T, Stark C, Holmbom J, Rosling A, Kuusilehto A, Tirri T (2010). Fate of bone marrow-derived stromal cells after intraperitoneal infusion or implantation into femoral bone defects in the host animal. J Tissue Eng.

[46] Meyerrose TE, De Ugarte DA, Hofling AA, Herrbrich PE, Cordonnier TD, Shultz LD (2007). In vivo distribution of human adipose-derived mesenchymal stem cells in novel xenotransplantation models. Stem Cells.

[47] Gao T, Yu Y, Cong Q, Wang Y, Sun M, Yao L (2018). Human mesenchymal stem cells in the tumour microenvironment promote ovarian cancer progression: the role of platelet-activating factor. BMC Cancer.

[48] Bazhanov N, Ylostalo JH, Bartosh TJ, Tiblow A, Mohammadipoor A, Foskett A (2016). Intraperitoneally infused human mesenchymal stem cells form aggregates with mouse immune cells and attach to peritoneal organs. Stem Cell Res Ther.

[49] Aboody KS, Brown A, Rainov NG, Bower KA, Liu S, Yang W (2000). Neural stem cells display extensive tropism for pathology in adult brain: evidence from intracranial gliomas. Proc Natl Acad Sci U S A.

[50] Aboody KS, Najbauer J, Metz MZ, D’Apuzzo M, Gutova M, Annala AJ (2013). Neural stem cell-mediated enzyme/prodrug therapy for glioma: preclinical studies. Sci Transl Med.

[51] Bergström M, Ivarsson ML, Holmdahl L (2002). Peritoneal response to pneumoperitoneum and laparoscopic surgery. Br J Surg.

[52] Oosterling SJ, van der Bij GJ, van Egmond M, van der Sijp JRM (2005). Surgical trauma and peritoneal recurrence of colorectal carcinoma. Eur J Surg Oncol.

