# Supplementary figures and images for: Allogeneic human neural stem cells for improved therapeutic delivery to peritoneal ovarian cancer
Source: Stem Cell Res Ther. 2021 Mar 24;12:205. doi: 10.1186/s13287-021-02226-8 (PMC7992793; doi:10.1186/s13287-021-02226-8)

**
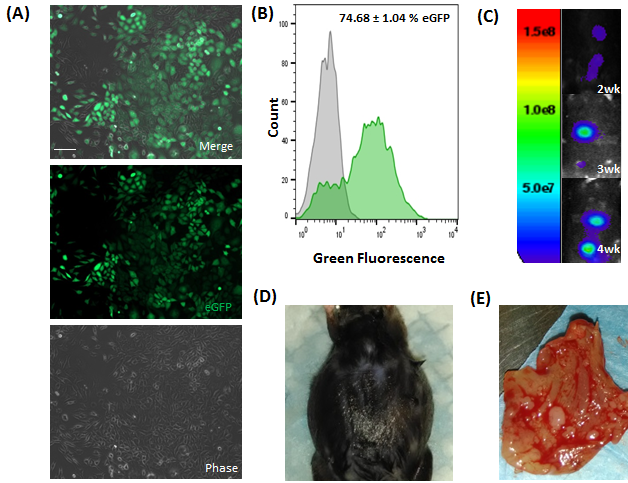
**

Supplement: Supplementary file 1 — Additional file 1: Supplementary Figure 1. Murine ID8 ovarian cancer cell line modified to stably express green fluorescent protein (eGFP) and firefly luciferase (ffluc). (A): Phase and fluorescence microscopy images of eGFP-positive ID8 (ID8.eGFP.ffluc) cells five days post-infection. Scale bar = 50 μm. (B): Representative histogram of ID8.eGFP.ffluc cells quantified by flow cytometry 15 days after infection. (C): Bioluminescent images confirming tumor engraftment after peritoneal administration of 5 × 10^6^ ID8.eGFP.ffluc cells into immunocompetent C57Bl/6 mice, color scale bar shown in relative light units. (D): Representative photograph demonstrating the development of ascites in a C57Bl/6 mouse, two months after inoculation with ID8.eGFP.ffluc cells. (E): Ex vivo photograph of the peritoneal wall harvested from a C57Bl/6 mouse inoculated with ID8.eGFP.ffluc cells. [file 13287_2021_2226_MOESM1_ESM.docx]

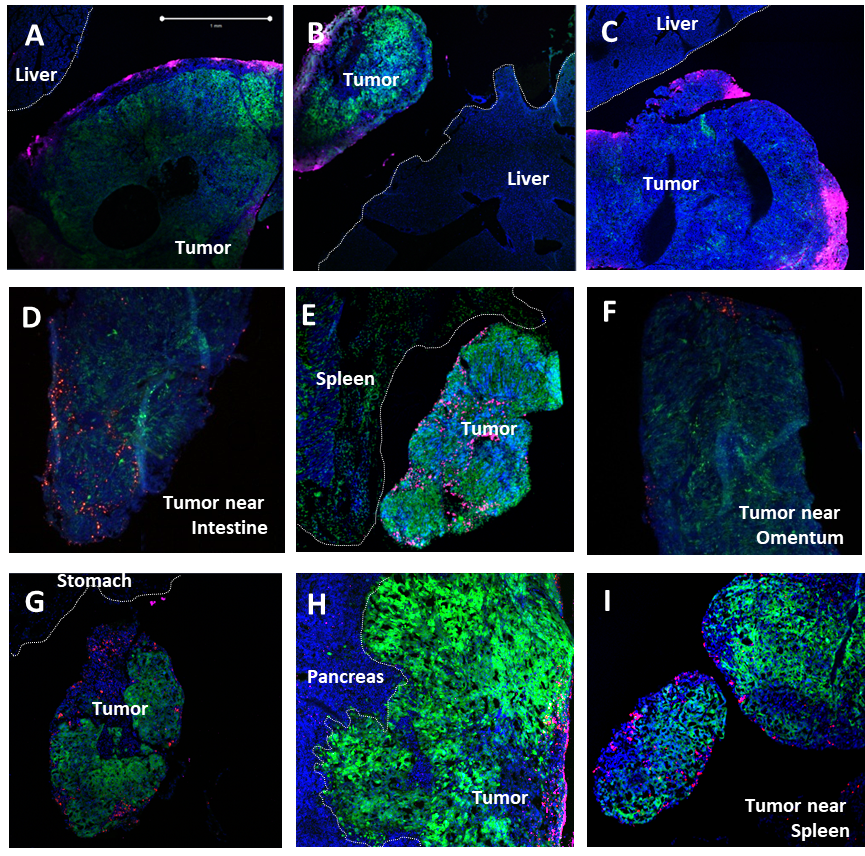

Supplement: Supplementary file 2 — Additional file 2: Supplementary Figure 2. Neural stem cells tropism to peritoneal ovarian cancer metastasis. Representative fluorescence images of neural stem cells labeled with either magenta DiD (A-C) or fluorescent orange (D-F) or red nanoparticles (G-I). Neural stem cells demonstrate good distribution in tumor but not in adjacent normal tissues (liver, intestine, kidney, omentum, stomach, pancreas or spleen). (2 million NSCs in 200uL PBS injected i.p. on Day 38; then harvested 4 days post-NSC injection). Scale bars = 1000 μm and applies to all images. [file 13287_2021_2226_MOESM2_ESM.docx]

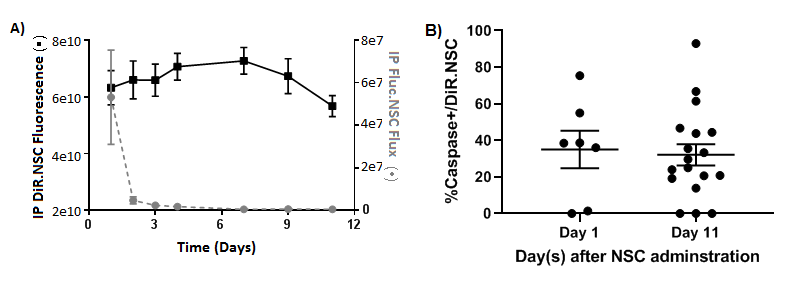

Supplement: Supplementary file 3 — Additional file 3: Supplementary Figure 3. NSC clearance kinetics. (A) Retention and viability of DiR-labeled NSC.eGFP.ffluc cells over a two-week period following IP injection into n = 4 tumor-bearing nude mice (solid line, DiR fluorescence; dotted line, ffluc expression). Once localized to tumors, the NSCs remained present for at least 3 days, according to steady NSC-associated DiR signals. However, NSC-specific firefly luciferase expression decrease quickly post-transplantation. (B) Fluorescent images of sectioned tumors obtained either 1 day (D1) or 11 days (D11) after IP injection of NSCs (magenta). Anti-caspase-3/7 antibodies were used to visualize apoptosis. DiR-labeled NSCs were ~ 30% positive for capsase-3/7 on both day 1 (35.0%) and day 11 (32.1%). Data represents % caspase + DIR-labeled NSCs observed in 3 stained slides of tumor sections over which 8 (Day 1) or 18 (Day 11) representative fields of view were quantified using ImageJ software. [file 13287_2021_2226_MOESM3_ESM.docx]
